# When does algorithmic complexity add causal value in environmental epidemiology?

**DOI:** 10.1097/EE9.0000000000000483

**Published:** 2026-04-23

**Authors:** Perla Simons, Salvador Diaz, Antonio Garcia Loureiro, Isaac Zablah

**Affiliations:** a Faculty of Medical Sciences, National Autonomous University of Honduras, Tegucigalpa, Honduras; b Department of Electronics and Computer Science, University of Santiago de Compostela, Santiago de Compostela, Spain

## To the Editor:

The short communication by Chen et al.^1^ delivers a rigorous comparative analysis of three causal estimators used to quantify mortality benefits associated with reductions in fine particulate matter with aerodynamic diameter ≤2.5 μm within a Canadian national cohort. Their central finding is that the targeted minimum loss-based estimation (TMLE) with SuperLearner, despite incorporating six candidate algorithms, produced a risk difference (−0.67 per 1,000 participants; 95% CI = −1.27, −0.06) statistically comparable to TMLE with parametric models (−0.83; 95% CI = −1.24, −0.43), which is striking. This concordance, coupled with substantially greater variance in the flexible estimator, highlights a structural condition of low-concentration, relatively homogeneous exposure settings where algorithmic complexity may not deliver meaningful inferential gains.

The author’s treatment of the bias-variance tradeoff is methodologically convincing. In contexts where the exposure-response relationship is smooth and the signal-to-noise ratio modest, ensemble learners often accumulate variance without achieving sufficient bias reduction.^2^ Their mean-squared error analysis reinforces this interpretation, showing that TMLE with SuperLearner surpasses parametric TMLE only within a narrow and implausible range of hypothetical true values. This finding, while persuasive, reminds us of the importance of contextualizing methodological conclusions rather than generalizing across diverse environmental settings.

Three dimensions deserve closer attention. First, exposure heterogeneity is decisive. In regions with broader fine particulate matter with aerodynamic diameter ≤2.5 μm distributions and steeper concentration gradients, such as South Asian or urban African environments where concentrations frequently exceed 40 μg/m^3^—adaptive algorithms may provide meaningful bias reduction that justifies the variance cost.^3^ To extrapolate Canadian findings to such contexts without empirical validation would be methodologically unsound.

Second, hyperparameter robustness demands caution. Although Chen et al^1^ tested 28 specifications with minimal variation, this stability likely reflects limited confounder dimensionality and uniform Canadian exposure.^4^ Personally, I see this as context-specific; in heterogeneous datasets, sensitivity could matter greatly, altering causal inference reliability.

Third, the most analytically instructive contrast lies not between TMLE specifications but between g-computation (−0.23; 95% CI = −0.46, 0.00) and both TMLE approaches. This divergence suggests that estimator choice exerts greater influence on causal estimates than algorithm selection, reinforcing the methodological priority of doubly robust frameworks.^2^ Personally, I find this hierarchy of importance particularly compelling, as it redirects attention from algorithmic novelty to estimator reliability.

**Figure 1.**
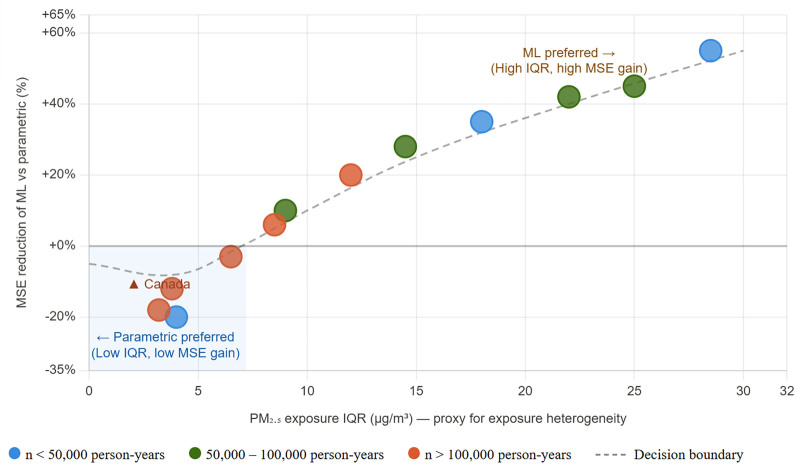
Conceptual framework for when algorithmic complexity may be more or less advantageous in environmental epidemiology. Note. This figure is intended as a qualitative conceptual schematic rather than an empirical estimate. Each point represents a hypothetical study setting positioned on an illustrative scale defined by exposure heterogeneity and expected relative estimator performance. The vertical placement of points was not estimated from the Canadian cohort data and should not be interpreted as an observed reduction in mean squared error. Rather, the figure heuristically summarizes the idea that flexible machine-learning approaches may become more attractive as exposure heterogeneity, sample support, and plausible nonlinearity increase, whereas simpler parametric approaches may remain preferable in lower-heterogeneity settings where gains in bias reduction are limited. The dashed curve represents a heuristic transition zone rather than a formally estimated decision boundary. The Canadian example is included only as an illustrative low-exposure, low-heterogeneity setting, consistent with the findings reported by Chen et al.^1^ SE, mean squared error; ML, machine learning; PM_2_.5, fine particulate matter with aerodynamic diameter ≤2.5 μm.

Future research should move beyond broad dichotomies between “machine learning” and “parametric” approaches. Instead, explicit decision criteria are needed to determine when flexible algorithms genuinely add inferential value. Such a framework should consider effective sample support, exposure heterogeneity, confounder dimensionality, and plausible nonlinearity.^5^ Without these criteria, algorithmic complexity risks being adopted as a default rather than a justified analytical decision, see Table 1. To aid interpretation, Figure 1 provides a conceptual schematic of how these features may shape the expected bias-variance tradeoff, rather than a quantitative decision rule.

**Table 1.** Proposed decision framework for selecting the analytical method in causal studies estimating the health effects of long-term PM_2.5_ exposure on mortality

Criterion	Favors parametric approach	Favors machine-learning approach
PM_2_._5_ exposure range	Narrow (≤8 μg/m^3^; near regulatory threshold)	Wide (>20 μg/m^3^; broad gradient across population)
Exposure heterogeneity	Low (uniform urban or national settings)	High (mixed urban-rural or transboundary pollution)
Effective sample size	Moderate (<50,000 person-years)	Large (>100,000 person-years)
Confounder dimensionality	Low (<15 baseline variables)	High (>30 variables with interactions)
Hypothesized nonlinearity	Mild or approximately linear	Pronounced (supra-linear or threshold effects)
Policy application context	Established national or regional standards	Local interventions or high-heterogeneity settings

All thresholds are approximate and should be adapted to the specific epidemiological context. This framework synthesizes methodological considerations from Chen et al.^1^ with general principles of doubly robust estimation^2,4^ and causal transportability.^5^

PM_2_._5_, fine particulate matter with aerodynamic diameter ≤2.5 μm.

Briefly, Chen et al^1^ contribute not only empirical estimates but also a methodological reflection that, in our view, is particularly valuable. Their findings show that in low exposure, homogeneous contexts, flexible algorithms may inflate variance without reducing bias. More broadly, the study underscores the need for context-sensitive frameworks that prioritize estimator choice and define clear criteria for algorithmic complexity. In that spirit, Figure 1 is intended only as a conceptual summary of the conditions under which added algorithmic flexibility may or may not be worthwhile in environmental epidemiology.

## Conflicts of interest statement

The authors declare that they have no conflicts of interest with regard to the content of this report.

## References

[1] Chen C Kaufman JS Rana J Benmarhnia T Chen H. Do we need flexible machine-learning algorithms to assess the effect of long-term exposure to fine particulate matter on mortality?: an example from a Canadian national cohort. Environ Epidemiol. 2025;9:e375. 40046729 10.1097/EE9.0000000000000375 PMC11882297

[2] Zivich PN Breskin A. Machine learning for causal inference: on the use of cross-fit estimators. Epidemiology. 2021;32:393–401. 33591058 10.1097/EDE.0000000000001332 PMC8012235

[3] Moccia C Moirano G Popovic M. Machine learning in causal inference for epidemiology. Eur J Epidemiol. 2024;39:1097–1108. 39535572 10.1007/s10654-024-01173-x PMC11599438

[4] Phillips RV Van Der Laan MJ Lee H Gruber S. Practical considerations for specifying a super learner. Int J Epidemiol. 2023;52:1276–1285. 36905602 10.1093/ije/dyad023

[5] Dahabreh IJ Haneuse SJA Robins JM. Study designs for extending causal inferences from a randomized trial to a target population. Am J Epidemiol. 2021;190:1632–1642. 33324969 10.1093/aje/kwaa270 PMC8536837

